# Preoperative nutritional risk index and postoperative one-year skeletal muscle loss can predict the prognosis of patients with gastric adenocarcinoma: a registry-based study

**DOI:** 10.1186/s12885-021-07885-7

**Published:** 2021-02-12

**Authors:** Kyung Won Kim, Koeun Lee, Jung-Bok Lee, Taeyong Park, Seungwoo Khang, Heeryeol Jeong, Chang-Seok Ko, Jeong-Hwan Yook, Byung-Sik Kim, In-Seob Lee

**Affiliations:** 1 Department of Surgery, Gastric Cancer Center, Asan Medical Center, University of Ulsan College of Medicine, 88, Olympic-ro 43-gil, Songpa-gu, Seoul, 05505 South Korea; 2 Department of Radiology and Research Institute of Radiology, Asan Medical Center, University of Ulsan College of Medicine, Seoul, South Korea; 3 Department of Nuclear Medicine, Asan Medical Center, University of Ulsan College of Medicine, Seoul, South Korea; 4 Division of Biostatistics, Center for Medical Research and Information, Asan Medical Center, University of Ulsan College of Medicine, Seoul, South Korea; 5 School of Computer Science and Engineering, Soongsil University, 369 Sangdo-Ro, Dongjak-Gu, Seoul, South Korea

**Keywords:** Gastric cancer, Surgery, Muscle loss, Nutrition, Prognosis, Survival, Sarcopenia

## Abstract

**Background:**

Patients with gastric cancer have an increased nutritional risk and experience a significant skeletal muscle loss after surgery. We aimed to determine whether muscle loss during the first postoperative year and preoperative nutritional status are indicators for predicting prognosis.

**Methods:**

From a gastric cancer registry, a total of 958 patients who received curative gastrectomy followed by chemotherapy for stage 2 and 3 gastric cancer and survived longer than 1 year were investigated. Clinical and laboratory data were collected. Skeletal muscle index (SMI) was assessed based on the muscle area at the L3 level on abdominal computed tomography.

**Results:**

Preoperative nutritional risk index (NRI) and postoperative decrement of SMI (dSMI) were significantly associated with overall survival (hazards ratio: 0.976 [95% CI: 0.962–0.991] and 1.060 [95% CI: 1.035–1.085], respectively) in a multivariate Cox regression analysis. Recurrence, tumor stage, comorbidity index were also significant prognostic indicators. Kaplan-Meier analyses exhibited that patients with higher NRI had a significantly longer survival than those with lower NRI (5-year overall survival: 75.8% vs. 63.0%, *P* <  0.001). In addition, a significantly better prognosis was observed in a patient group with less decrease of SMI (5-year overall survival: 75.7% vs. 66.2%, *P* = 0.009). A logistic regression analysis demonstrated that the performance of preoperative NRI and dSMI in mortality prediction was quite significant (AUC: 0.63, *P* <  0.001) and the combination of clinical factors enhanced the predictive accuracy to the AUC of 0.90 (*P* <  0.001). This prognostic relevance of NRI and dSMI was maintained in patients experiencing tumor recurrence and highlighted in those with stage 3 gastric adenocarcinoma.

**Conclusions:**

Preoperative NRI is a predictor of overall survival in stage 2 or 3 gastric cancer patients and skeletal muscle loss during the first postoperative year was significantly associated with the prognosis regardless of relapse in stage 3 tumors. These factors could be valuable adjuncts for accurate prediction of prognosis in gastric cancer patients.

**Supplementary Information:**

The online version contains supplementary material available at 10.1186/s12885-021-07885-7.

## Background

Surgery is the mainstay of curative treatment for gastric cancer. Gastrectomy induces physiologic derangements including worsened nutritional status, significant weight loss, and decreased muscle and fat volume. The loss of stomach reservoir function, rapid intestinal transit time, and foods bypassing the proximal small intestine are responsible for these postoperative changes [1–3].

In the first year after gastrectomy, most patients experience dramatic physiologic changes, like cascade of sarcopenia and malnutrition. Indeed, our prior study demonstrated that skeletal muscle mass and nutritional parameters decrease sharply for the first 3 months and slowly decrease for the remaining 9 months, eventually leading to a loss of 8 ~ 15% of the initial body weight and 3 ~ 5% of the muscle area [3]. In addition, adjuvant chemotherapy, which is the standard treatment in stage 2 and 3 gastric cancer, exacerbates skeletal muscle loss and nutritional status of patients [4].

Several tumor factors including the TNM staging system are powerful predictors of survival in gastric cancer patients treated with surgery; however, it is insufficient in reflecting the heterogeneity of the clinical course. A number of patient variables such as age, performance status, and neutrophil/lymphocyte ratio have been identified as prognostic factors for overall survival in the last decade [5–7]. Recently, the prognostic value of preoperative sarcopenia and nutritional status have been gaining emphasis [8, 9]. However, we have been impressed that progressive skeletal muscle loss after gastrectomy might also be a powerful prognostic indicator based on thousands of cases of practice in our dedicated gastric cancer center. Especially, it seems that the muscle loss at 1 year after surgery, when adjuvant chemotherapy is completed, might be particularly important to indicate overall health state and prognosis of patients.

Thus, we hypothesized that the progressive muscle loss during the first postoperative year is a significant predictor of overall survival along with preoperative nutritional status in patients with stage 2 or 3 gastric cancer who survived longer than 1 year. To evaluate our hypothesis, we performed this large-scale study with the aid of artificial intelligence techniques to measure body composition.

## Methods

The protocol for this retrospective and registry-based cohort study was approved by the institutional review board of Asan Medical Center, Seoul, Korea (IRB No. 2017–0216). This study was reported according to the Transparent Reporting of a multivariable prediction model for Individual Prognosis or Diagnosis (TRIPOD) guidelines [10].

### Patient selection

The current study was conducted on the basis of a comprehensive and prospectively built gastric cancer surgery registry containing the demographic characteristics of patients, preoperative evaluation results, surgery-related and postoperative outcomes, pathologic information, and follow-up data. From the registry, the data of 9940 patients who received surgery for biopsy-proven primary gastric adenocarcinoma from 2007 to 2012 at Asan Medical Center, Seoul, Korea were initially extracted. Subsequently, we included patients based on specific inclusion and exclusion criteria.

**Fig. 1:**
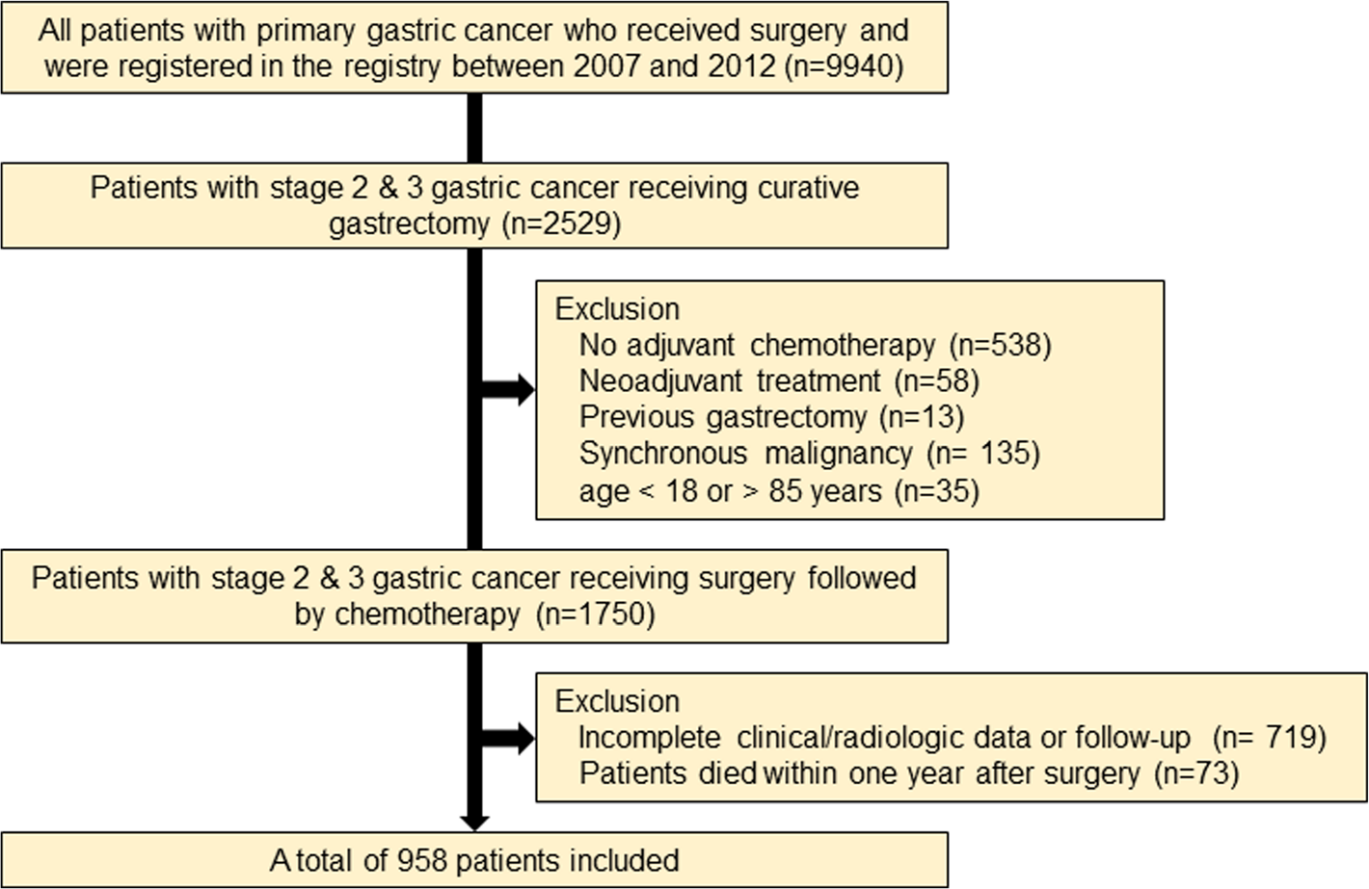
Flow chart of the patient selection

The inclusion criteria were as follows: (a) patients who were treated with curative gastrectomy followed by adjuvant chemotherapy using S-1 or capecitabine plus oxaliplatin (XELOX) for pathologic stage 2 and 3 gastric cancer based on the American Joint Committee on Cancer 7th edition [11], (b) patients aged between 18 and 85 years, (c) patients who abide by a regular follow-up protocol with available data on demographic measures, laboratory findings, and abdominopelvic computed tomography (CT) images. Patients (a) who died within 1 year after surgery, (b) who received neoadjuvant treatment, (c) with a history of previous partial gastrectomy, (d) with any synchronous malignancy in another organ, and (e) with inappropriate clinical or radiologic data were excluded. The patient selection process is illustrated in Fig. 1.

### Medical data collection and follow-up

The preoperative clinicopathologic characteristics of the patients, including age, sex, body weight, height, body mass index (BMI), and history of any synchronous malignancy or comorbidity, were evaluated. Comorbidity was assessed by Charlson Comorbidity Index [12]. Information about the type of operation (open vs. laparoscopic approach), type of gastrectomy (distal vs. total gastrectomy), extent of lymph node dissection (D1 vs. D1+ vs. D2), and pathologic data including Lauren’s classification [13] and pathologic tumor stage were collected from the registry.

Laboratory parameters such as serum protein and albumin at preoperative and postoperative periods were extracted from the registry. The nutritional risk index (NRI) was calculated based on the formula (1.519 × serum albumin g/L) + 0.417 × (present weight / usual weight) × 100 [14]. The difference in NRI (dNRI) between before and 1 year after surgery was assessed. Body weight and BMI were measured at all visits. Recurrence was evaluated by serum tumor marker measurement, endoscopy, and abdominopelvic CT following the guidelines for gastric cancer treatment [15].

### Assessment of body composition

CT scans obtained before and 1 year after surgery were selected for body morphometric analysis. Preoperative CT was checked within 1 month before surgery on average. Body composition was assessed with abdominopelvic CT using an automated artificial intelligence software (AID-U™, iAID Inc., Seoul, Korea), which was developed using a fully convolutional network (FCN) segmentation technique [16]. A specialized abdominal radiologist (K.W.K), who was blinded to the clinical information, selected the axial CT slice at the L3 vertebral inferior endplate level in a semi-automatic manner with the aid of coronal reconstructed images. Then, the chosen images were automatically segmented to generate the boundary of total abdominal muscles. The skeletal muscle area (SMA) including all muscles on the selected axial images, i.e., psoas, paraspinal, transversus abdominis, rectus abdominis, quadratus lumborum, and internal and external obliques, were demarcated using predetermined thresholds (− 29 to + 150 Hounsfield units). The visceral fat area (VFA) and subcutaneous fat area (SFA) were also demarcated using fat tissue thresholds (− 190 to − 30 Hounsfield units) (Supplementary Figure S1). The SMA was adjusted for the square of the height (SMA/height^2^), which is referred to as the skeletal muscle index (SMI) [17]. The differences in SMA and SMI between before and 1 year after surgery were calculated to yield dSMA and dSMI, respectively.

### Statistical analysis

Continuous data were reported as means with standard deviations, and categorical data were presented as proportions. Normality was assessed using frequency histograms and the Kolmogorov-Smirnov test. A paired t-test was used to compare preoperative with postoperative body composition and nutritional parameters. Overall survival was defined as the time interval between the date of surgery and the date of patient’s death from any cause. Patients were censored at 5 years for overall survival if they were alive at 5 years after surgery. Follow-up loss and recurrence were also regarded as censoring.

The primary endpoint of this study was to determine prognostic factors to predict overall survival in patients who lived longer than 1 year after gastrectomy. A Cox proportional hazards model was used for univariate and multivariate analyses, and outcomes were reported as hazards ratios and 95% confidence intervals. The covariates that were significant in univariate Cox analysis were included in multivariate one. In multivariate analysis, the backward elimination method was used to fit the model and to determine the final significant predictors after confirming that there was no significant multicollinearity between variables by examining the correlation matrices.

The secondary endpoint was to evaluate predictive performance of the progressive muscle loss (dSMI) and preoperative nutritional status (NRI) to predicting mortality. Patients were dichotomized (high-risk vs. low-risk group) according to the median value of dSMI and preoperative NRI. Survival curves were estimated for each group using the Kaplan-Meier method and compared statistically using the log rank test. Logistic regression analysis with Enter model was carried out to assess the performance dSMI and NRI for prediction of patient mortality during the follow-up period, and the performance was evaluated with receiver operating characteristic (ROC) curves and area under the curves (AUCs).

As a subgroup analysis, all these statistical analyses were performed in a recurrence group and additional analyses were also undertaken according to tumor stage. *P*-value of < 0.05 was used as the level of significance for the study. Statistical analyses were performed using SAS software version 9.4 (SAS Institute Inc., Cary, NC, USA) and IBM SPSS® version 26 (IBM, Armonk, NY, USA).

## Results

### Baseline characteristics

Figure 1 illustrates the patient selection process. Among the initial 9940 patients who were registered in the gastric cancer surgery registry, there were 1750 patients with stage 2 or 3 gastric adenocarcinoma who were treated with gastrectomy followed by adjuvant chemotherapy. During the process, we excluded 538 patients not receiving adjuvant treatment including cases with tumor stage of pT1N+ or pT3N0 (*n* = 455) which are not indicated to adjuvant treatment according to Korean and Japanese gastric cancer treatment guidelines, and those with poor general condition, severe comorbidity, and postoperative complication (*n* = 83).

After excluding those who had insufficient image quality for body morphometry analysis or incomplete follow-up data (*n* = 719) and who died within 1 year after surgery (*n* = 73), a total of 958 patients were included in the study. As seen in Table 1, we divided patients into a recurrence group (*n* = 293) and a non-recurrence group (*n* = 665), because the treatment and prognosis of two population differ significantly. In a recurrence group, 26 patients experienced tumor relapse within 6 months after surgery. Type of gastrectomy and distribution of tumor stage were different between two groups; total gastrectomy and tumors with an advanced stage were more frequently observed in patients experiencing relapse. The other variables did not differ significantly between the two groups.

**Table 1:** Clinicopathologic characteristics of patients with stage 2 and 3 gastric cancer

Variables	Whole patients (***N*** = 958)	Recurrence group (***N*** = 293)	Non-recurrence group (***N*** = 665)	***P***-value
Mean age at operation, years (±SD)	57.0 (±12.3)	57.7 (±12.6)	56.7 (±12.2)	0.240
Sex				0.449
Male	624 (65.1%)	196 (66.9%)	428 (64.4%)	
Female	334 (34.9%)	97 (33.1%)	237 (35.6%)	
Comorbidity				0.763
Diabetes	138 (14.4%)	44 (15.0%)	94 (14.1%)	
Hypertension	271 (28.3%)	86 (29.4%)	185 (27.8%)	
Lung disease	38 (4.0%)	14 (4.8%)	24 (3.6%)	
Cerebrovascular accident	64 (6.7%)	20 (6.8%)	44 (6.6%)	
Heart disease	25 (2.6%)	8 (2.7%)	17 (2.6%)	
Liver cirrhosis	9 (0.9%)	4 (1.4%)	5 (0.7%)	
Renal disease	15 (1.6%)	4 (1.4%)	11 (1.7%)	
Charlson Comorbidity Index				0.065
0	243 (24.4%)	62 (21.1%)	181 (27.2%)	
1–2	465 (48.5%)	147 (50.2%)	318 (47.8%)	
3–6	250 (26.1%)	84 (28.7%)	166 (25.0%)	
Type of operation				0.063
Laparoscopy	102 (10.6%)	23 (7.8%)	79 (11.9%)	
Open	856 (89.4%)	270 (92.2%)	586 (88.1%)	
Type of gastrectomy				**0.007**
Distal gastrectomy	549 (57.2%)	149 (50.9%)	400 (60.2%)	
Total gastrectomy	409 (42.8%)	144 (49.1%)	265 (39.8%)	
Extent of lymphadenectomy				0.934
D1	20 (2.0%)	6 (2.0%)	14 (2.1%)	
D1 plus	241 (25.2%)	77 (26.3%)	164 (24.7%)	
D2	697 (72.8%)	210 (71.7%)	487 (73.2%)	
TNM stage^a^				**< 0.001**
2A	199 (20.8%)	26 (8.9%)	173 (26.0%)	
2B	237 (24.7%)	61 (20.8%)	176 (26.5%)	
3A	194 (20.3%)	47 (16.0%)	147 (22.1%)	
3B	194 (20.3%)	80 (27.3%)	114 (17.1%)	
3C	134 (14.0%)	79 (27.0%)	55 (8.3%)	

^a^TNM stage was based on the American Joint Committee on Cancer 7th edition

Among patients, 42.4% had at least one comorbidity and 89.4% received open surgery. Distal gastrectomy was more frequently performed than total gastrectomy (57.2% versus 42.8%) (Table 1).

### Changes in body composition and nutritional parameters

Body weight diminished during the first postoperative year (mean loss of 5.9 kg). All parameters related to body composition (SMA, SMI, SFA, VFA) and NRI measured at 1 year after surgery showed a significant decrement compared with preoperative values as well (Supplementary Table S1). These changes were consistently observed in both the recurrence group and the non-recurrence group. The representative cases were presented in Supplementary Figure S2.

### Prognostic relevance of skeletal muscle loss and NRI

Univariate and multivariate cox-hazard regression analysis results were presented in Table 2. In univariate analysis, the recurrence was the strongest prognostic indicator (HR 13.992). Among the clinicopathologic variables, old age, male sex, higher comorbidity index, advanced tumor stage, open surgery, total gastrectomy, and larger tumor size were associated with a shorter survival period in patients with stage 2 and 3 gastric cancer. Among preoperative body composition and nutrition related parameters, SFA and NRI affected prognosis. Among the parameters for body composition and nutrition change between before and 1 year after surgery, dSMI was predictive of overall survival.

**Table 2:** Cox proportional hazards regression analyses of overall survival according to tumor recurrence

Variables	Whole group (***N*** = 958)						Recurrence group (***N*** = 293)					
	Univariate analysis			Multivariate analysis			Univariate analysis			Multivariate analysis		
	HR	95% CI	***P-***value	HR	95% CI	***P-***value	HR	95% CI	***P-***value	HR	95% CI	***P-***value
**Clinicopathologic**												
Age (continuous)	1.019	1.011–1.028	**< 0.001**				1.002	0.992–1.012	0.701			
Sex (male)	1.242	1.007–1.531	**0.043**				1.009	0.785–1.297	0.943			
BMI (continuous)	0.968	0.935–1.001	0.061				0.999	0.960–1.039	0.955			
CCI (continuous)	1.223	1.138–1.314	**< 0.001**	1.173	1.082–1.272	**< 0.001**	1.068	0.977–1.169	0.150			
TNM stage (stage 3)	1.927	1.573–2.362	**< 0.001**	1.324	1.054–1.663	**0.016**	1.528	1.175–1.987	**0.002**	1.312	0.998–1.724	0.051
Operation type (open)	1.458	1.027–2.072	**0.035**				1.308	0.845–2.024	0.229			
Gastrectomy type (total)	1.240	1.020–1.506	**0.030**				1.109	0.875–1.405	0.393			
Tumor size (continuous)	1.079	1.052–1.108	**< 0.001**				1.022	0.990–1.055	0.173			
Tumor location (upper vs. others)	1.058	0.949–1.181	0.310				1.033	0.921–1.159	0.575			
Lauren’s classification (other types vs. intestinal)	0.929	0.814–1.060	0.272				1.245	1.073–1.445	**0.004**	1.157	0.989–1.354	0.069
Recurrence	13.992	11.197–17.486	**< 0.001**	16.838	13.089–21.662	**< 0.001**	NA	NA	NA	NA	NA	NA
**Preop. Body/Nutrition** (as a continuous variable)												
SMI	1.000	0.994–1.006	0.965				1.001	0.995–1.007	1.001			
SFA	0.997	0.995–0.999	**< 0.001**	0.998	0.996–1.000	0.075	0.999	0.997–1.001	0.497			
VFA	1.000	0.998–1.002	0.864				0.999	0.997–1.001	0.447			
NRI	0.960	0.948–0.973	**< 0.001**	0.976	0.962–0.991	**0.002**	0.981	0.966–0.996	**0.014**	0.966	0.950–0.983	**< 0.001**
**Body/Nutrition change** (as a continuous variable)												
dSMI	1.043	1.019–1.066	**< 0.001**	1.060	1.035–1.085	**< 0.001**	1.045	1.019–1.071	**< 0.001**	1.045	1.019–1.073	**< 0.001**
dSFA	1.000	0.998–1.003	0.816				1.004	1.002–1.007	**0.002**	1.005	1.002–1.008	**0.002**
dVFA	1.000	0.998–1.002	0.881				1.001	0.998–1.003	0.660			
dNRI	1.000	0.993–1.006	0.917				1.004	0.995–1.002	0.406			

*Abbreviations*: *HR* Hazards ratio, *CI* Confidence interval, *CCI* Charlson comorbidity index, *Preop* Preoperative, *SMA* Skeletal muscle area, *SMI* Skeletal muscle index, *SFA* subcutaneous fat area, *VFA* Visceral fat area, *NRI* Nutritional risk index, *dSMA* Difference in SMA between before and one year after surgery, *dSMI* Difference in SMI between before and one year after surgery, *dNRI* Difference in NRI between before and one year after surgery

When these significant variables were included in the multivariate analysis as covariates, the recurrence was also the strongest predictor of overall survival (HR 16.838), and comorbidity index, TNM stage, preoperative NRI, and dSMI remained as significant prognostic factors. These results support our hypothesis that progressive muscle loss during the first year after gastrectomy is an independent predictor of worse prognosis (dSMI; HR 1.060) along with protective effect of preoperative nutritional status (NRI; HR 0.976).

As tumor recurrence was a dominant factor, we separately investigated the prognostic relevance of muscle loss in the recurrence group. In univariate analysis, in addition to tumor stage, Lauren’s classification, and preoperative NRI, dSMI, and dSFA were related with survival. Notably, in multivariate analysis, the dSMI (HR 1.045) and NRI (HR 0.966) maintained their significances as independent prognostic indicators of overall survival along with pathologic tumor stage and dSFA. These analyses enabled us to ascertain that dSMI and NRI were consistently associated with the prognosis irrespective of tumor recurrence. Clinicopathologic characteristics based on these two indicators were compared in Supplementary Table S2.

### Prognostic impact of NRI and skeletal muscle loss on stage 3 gastric adenocarcinoma

Next, as the tumor stage was an independent prognostic factor, we further undertook a subgroup analysis by tumor stage (Table 3). A multivariate analysis exhibited that NRI was a consistent prognostic indicator for overall survival regardless of tumor stage. Also, in patients with stage 3 gastric cancer, dSMI was significantly associated with the prognosis along with tumor recurrence, comorbidity index, and NRI in multivariate analysis. However, the statistical significance of muscle loss was not observed in stage 2 tumors.

**Table 3:** Cox proportional hazards regression analyses of overall survival according to tumor stage

Variables	Stage 2 (***N*** = 436)						Stage 3 (***N*** = 522)					
	Univariate analysis			Multivariate analysis			Univariate analysis			Multivariate analysis		
	HR	95% CI	***P-***value	HR	95% CI	***P-***value	HR	95% CI	***P-***value	HR	95% CI	***P-***value
**Clinicopathologic**												
Age (continuous)	1.028	1.013–1.044	**< 0.001**				1.016	1.006–1.027	**0.002**			
Sex (male)	1.232	0.868–1.749	0.243				1.224	0.943–1.590	0.130			
BMI (continuous)	0.981	0.924–1.041	0.516				0.975	0.935–1.016	0.220			
CCI (continuous)	1.386	1.227–1.566	**< 0.001**	1.154	1.016–1.310	**0.028**	1.161	1.063–1.268	**< 0.001**	1.335	1.132–1.573	**< 0.001**
Operation type (open)	1.022	0.659–1.584	0.924				1.644	0.874–3.093	0.123			
Gastrectomy type (total)	1.190	0.851–1.664	0.309				1.139	0.895–1.449	0.290			
Tumor size (continuous)	1.075	1.023–1.130	**0.005**				1.061	1.028–1.096	**< 0.001**			
Tumor location (upper vs. others)	1.024	0.842–1.246	0.814				1.072	0.942–1.220	0.293			
Lauren’s classification (other types vs. intestinal)	0.865	0.685–1.092	0.224				0.915	0.780–1.073	0.275			
Recurrence	14.203	10.027–20.118	**< 0.001**	12.748	8.946–18.167	**< 0.001**	12.486	9.275–16.808	**< 0.001**	17.841	12.542–25.378	**< 0.001**
**Preop. Body/Nutrition** (as a continuous variable)												
SMI	0.998	0.987–1.010	0.801				1.000	0.995–1.007	0.746			
SFA	0.997	0.993–0.999	**0.036**	0.997	0.994–0.999	**0.044**	0.997	0.995–0.999	**0.008**			
VFA	1.000	0.997–1.003	0.961				1.000	0.998–1.002	0.866			
NRI	0.957	0.936–0.978	**< 0.001**	0.971	0.948–0.995	**0.019**	0.968	0.953–0.983	**< 0.001**	0.973	0.956–0.991	**0.004**
**Body/Nutrition change** (as a continuous variable)												
dSMI	1.011	0.967–1.057	0.619				1.038	1.013–1.065	**0.003**	1.042	1.015–1.069	**0.002**
dSFA	1.001	0.997–1.005	0.536				0.999	0.996–1.002	0.614			
dVFA	1.001	0.997–1.005	0.518				0.999	0.996–1.002	0.367			
dNRI	1.001	0.992–1.011	0.784				1.008	0.998–1.019	0.103			

*Abbreviations*: *HR* Hazards ratio, *CI* Confidence interval, *CCI* Charlson comorbidity index; Preop., preoperative, *SMA* Skeletal muscle area, *SMI* Skeletal muscle index, *SFA* subcutaneous fat area, *VFA* Visceral fat area, *NRI* Nutritional risk index, *dSMA* Difference in SMA between before and one year after surgery, *dSMI* Difference in SMI between before and one year after surgery, *dNRI* Difference in NRI between before and one year after surgery

### Performance of skeletal muscle loss and NRI as prognostic stratifiers

**Fig. 2:**
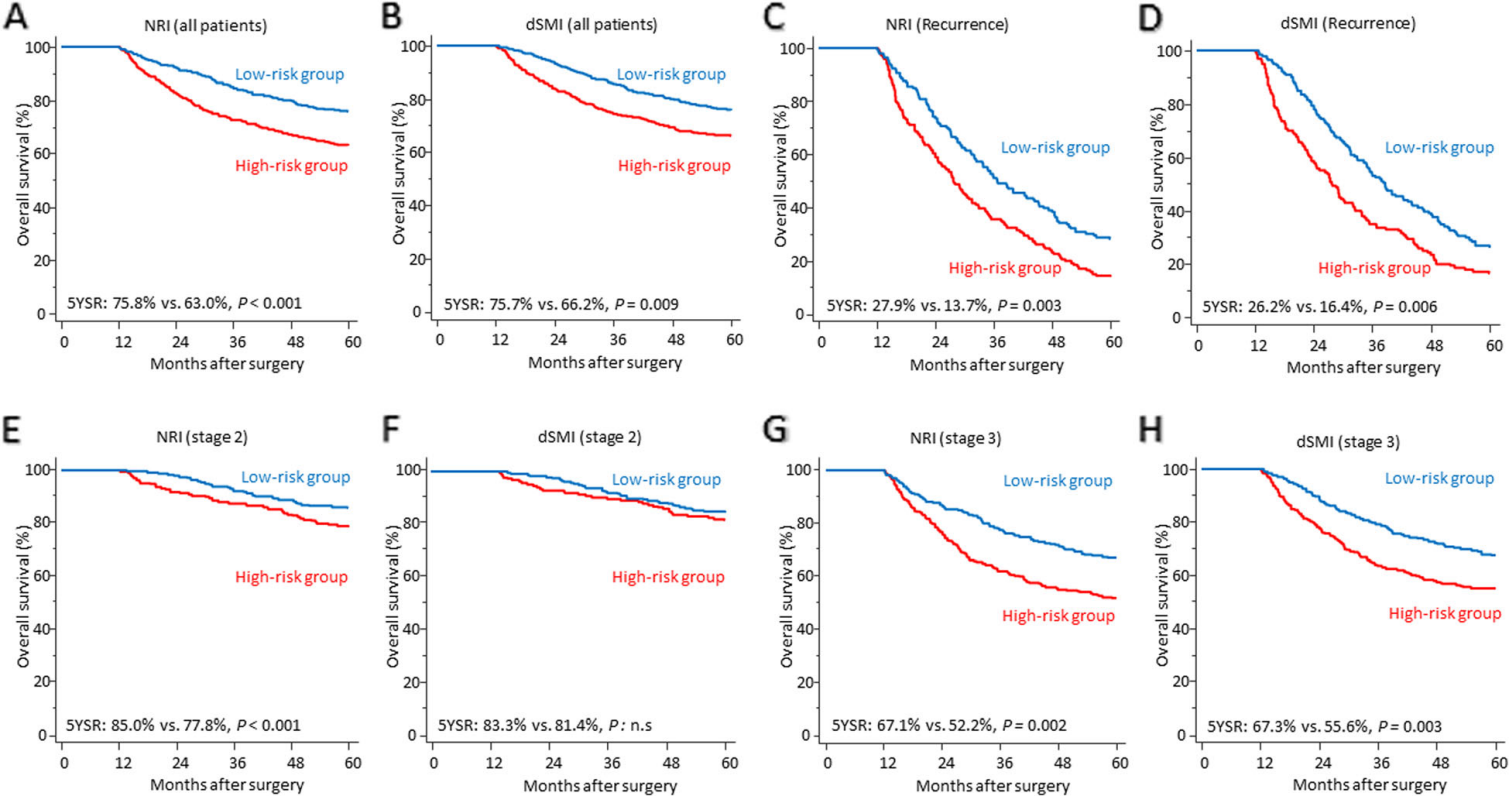
Kaplan-Meier curves showing the survival differences between two risk groups defined by NRI and skeletal muscle loss. In the whole patient group, patients were dichotomized by the median value of NRI (**a**) and dSMI (**b**). A subgroup analysis was undertaken in the recurrence group and patients were divided according to NRI (**c**) and dSMI (**d**). By pathologic tumor stage, analyses were performed by two variables in stage 2 (**e** and **f**) and stage 3 (**g** and **h**) gastric cancer patients

The 5-year overall survival rate of patients included in this study were 69.4%. To evaluate the role as a prognostic stratifier, patients were dichotomized (high-risk vs. low-risk group) according to the median value of preoperative NRI (100.941) and dSMI (− 2.059). Kaplan-Meier curves showed that patients with a better nutritional status (higher NRI) had a significantly better prognosis (5-year overall survival rate: 75.8% vs. 63.0%, *P* <  0.001) (Fig. 2a). In addition, patients with less decrease of skeletal muscle (smaller dSMI) also demonstrated longer survival period (5-year overall survival rate: 75.7% vs. 66.2%, *P* = 0.009) (Fig. 2b). In a subgroup analysis with the relapse group, a significant survival difference was observed between high-risk group and low-risk group for both NRI (5-year overall survival rate: 27.9% vs. 13.7%, *P* = 0.003) and dSMI (26.2% vs. 16.4%, *P* = 0.006) (Fig. 2c and d). Then, we conducted survival analyses based on tumor stage and it demonstrated that NRI stratified the prognosis in stage 2 group (85.0% vs. 77.8%, *P* <  0.001) (Fig. 2e) but dSMI was not associated with the survival difference (Fig. 2f). However, we again ascertained that both factors significantly divided the survival curves of patients with stage 3 gastric cancer in line with Cox regression analysis (Fig. 2g and h).

**Fig. 3:**
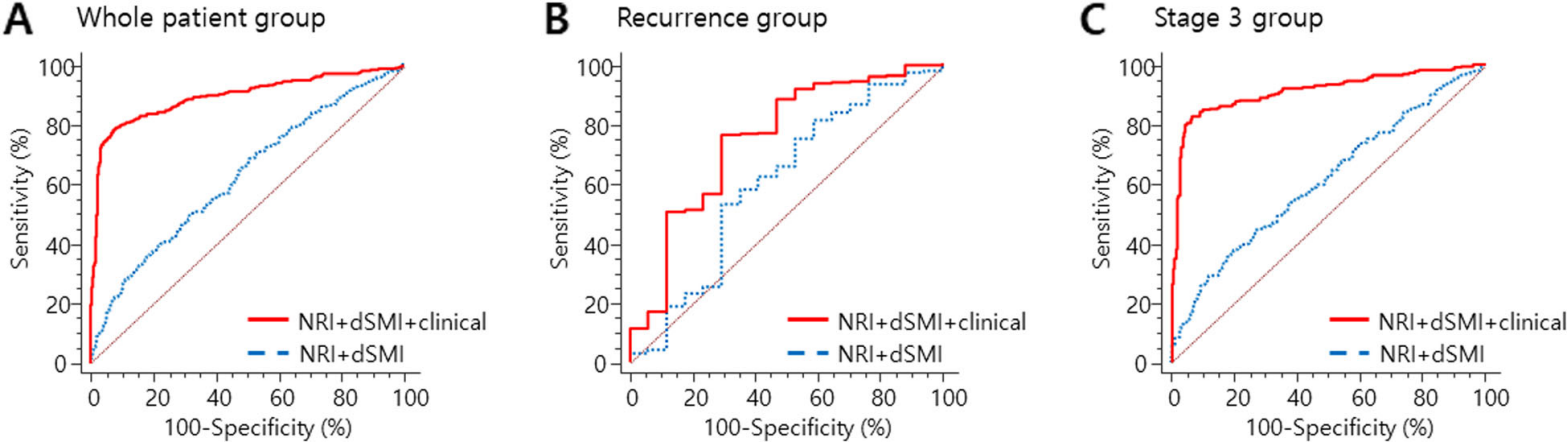
Receiver operating characteristic (ROC) curves demonstrating the performance of preoperative NRI and dSMI in predicting mortality and the enhanced performance of prediction was seen when combined with clinical factors in the whole group (**a**), the recurrence group (**b**), and stage 3 gastric cancer patient group (**c**)

Next, we evaluated two variables’ performance in mortality prediction with a logistic regression model. For all patients with stage 2 and 3 gastric cancer, the accuracy to predict mortality resulted in the AUC of 0.61 (95% CI: 0.58–0.64, *P* <  0.001) and 0.54 (95% CI: 0.51–0.57, *P* = 0.015) in NRI and dSMI, respectively. Then, we assessed a combined prediction model consisting of two factors and it exhibited the AUC of 0.63 (*P* <  0.001), which was higher than an individual variable. Subsequently, clinical factors including tumor recurrence, stage, and comorbidity index which were significant in multivariate analysis were combined to the prediction model, and the AUC was increased to 0.90 (95% CI: 0.88–0.92, sensitivity 79.6%, specificity 91.9%, *P* <  0.001) (Fig. 3a). In the recurrence group, although the individual performance of NRI and dSMI was not significant, a combined model with staging and Lauren’s classification exhibited the AUC of 0.75 and it was statistically significant (95% CI: 0.69–0.80, sensitivity 76.3%, specificity 70.6%, *P* = 0.005) (Fig. 3b). Finally, we evaluated the performance of two variables in stage 3 tumors and yielded the AUC of 0.61 but, when combined with recurrence and comorbidity index, the performance was improved to the AUC of 0.91 (95% CI: 0.88–0.94, sensitivity 82.8%, specificity 93.5%, *P* <  0.001) (Fig. 3c).

## Discussion

In this study, the multivariate Cox-hazard regression results supported our hypothesis that the one-year loss of skeletal muscle after gastrectomy (dSMI) is a significant predictor of overall survival along with preoperative nutritional status (NRI) in gastric cancer patients who survived longer than 1 year. Although several powerful prognostic factors for overall survival including recurrence, TNM stage, and comorbidity (HRs, 16.838, 1.324, and 1.173, respectively) were included in the multivariate analysis, the dSMI (HR 1.060) and preoperative NRI (HR 0.976) eventually remained as independent predictors. Kaplan-Meier curves also showed that less degree of postoperative muscle loss (small dSMI) and a better nutritional status before surgery (NRI) had a protective effect on the survival. These prognostic values of two variables were maintained even in patients with relapsed tumor and underscored in stage 3 gastric adenocarcinoma.

Nowadays, progressive loss of skeletal muscle mass has been highlighted as a prognostic factor in cancer patients, which is associated with cancer cachexia. Indeed, cachexia significantly contributes to mortality in patients with malignancy, accounting for more than 20% of cancer deaths [18]. Especially, in patients with gastric cancer, sarcopenia is known to be highly prevalent [19, 20], and a marked reduction in the initial body weight and muscle mass during the first postoperative year closely mimics the malnutrition and cancer cachexia cascade. Although several studies have reported preoperative sarcopenia as an indicator of poor prognosis, it could not reflect the prognostic uniqueness of the steep deterioration of muscle amount after gastrectomy [17, 21]. The increased mortality related to muscle loss in this study might be explained by that a higher degree of cachexia aggravates systemic inflammation and metabolic alterations leading to the poor prognosis in combination with a decrease in body protein stores. In addition, loss of muscle mass might also influence the tolerability of chemotherapy in those patients.

Our study, as a large-scale research, demonstrated that progressive muscle loss during the first year after gastrectomy is also a significant indicator of worse prognosis. Indeed, in a recent study based on a randomized multicenter trial, a marked loss in muscle or subcutaneous/visceral fat at 6 months after surgery could predict poor prognosis in patients with stage 2/3 gastric cancer [22]. However, some limitations such as relatively small number of cases, inclusion of a significant number of patients without adjuvant treatment within the study cohort, and the absence of subgroup analyses alleviated the importance of the prognostic potential of muscle loss. To overcome these shortcomings, we confined our study group to patients undergoing adjuvant chemotherapy and performed several subgroup analyses by the presence of tumor recurrence and stage information. As a result, we were able to reveal the valuable prognostic relevance of NRI in stage 2 and 3 gastric cancers and muscle loss in stage 3 tumors.

As a nutritional factor, we adopted the NRI among various indices in this study because it consists of objective and easily measurable parameters used for follow-up of gastric cancer in outpatient settings and does not require additional measurement (e.g., triceps skin fold, detailed laboratory variable such as C-reactive protein, glycoprotein, prealbumin, and neutrophil/lymphocyte count). The preoperative NRI revealed its prognostic value in the recurrence group as well as whole patients. This result coincides with prior studies demonstrating that preoperative malnutrition could influence cancer-related or -unrelated death in malignancies [23, 24]. However, the change in NRI between before and 1 year after surgery (dNRI) did not influence the overall survival in gastric cancer patients and the level of albumin was higher at postoperative 1 year compared to preoperative value. It might be attributed to an active educational program emphasizing high-protein diet to avoid dumping syndrome and to improve nutritional state after surgery, or enhanced adaptation of small intestine for protein absorption. However, our results should not hamper the importance of nutritional support for patients with gastric cancer.

Among several prognostic factors which were significant in this study, the dSMI and preoperative NRI could be valuable indicators because they may have potentials to improve prognosis through efforts to enhance the nutritional status before surgery or maintain the muscle mass with intensive exercise and nutritional support after gastrectomy. Recently, exercise and physical activities during cancer treatment has been greatly emphasized in various malignancies, and the therapeutic benefit of exercise interventions on cancer patients have been investigated [25–27]. In addition, in 2020, the American Cancer Society guidelines has been issued for diet and physical activity for cancer patients [28].

Only patients with stage 2 and 3 gastric cancer were included in the study because there is a discrepancy in treatment strategy and prognosis between stage 1 tumors and more advanced cancers. The vast majority of patients with stage 1 gastric cancers are treated with surgery alone and have an excellent prognosis of 5-year overall survival rate reaching to 95%. However, stage 2 and 3 cancers are indicated to surgery followed by chemotherapy, yielding the 3-year overall survival rate of 80.0%, and a significant number of patients experience relapse despite of adjuvant treatment [29, 30]. In this perspective, the prognostic implication of body composition and nutrition becomes higher in stage 2 and 3 gastric cancer.

We acknowledge that this study has some limitations. First, although data were collected prospectively in the registry, this is a retrospective study based on data from a single institution. Second, as we excluded patients who died within 1 year postoperatively, the prognostic effect of skeletal muscle loss was applicable to patients who survived longer than 1 year after surgery. Third, we did not consider chemotherapy induced toxicity on the survival outcomes. Fourth, despite prognostic relevance, the therapeutic benefit of efforts to support nutrition and preserve muscle mass was not proven. Finally, as our study excluded patients treated with neoadjuvant strategy for advanced gastric cancer, clinical significance is limited in patients from Western countries. To overcome these limitations, a well-designed prospective multi-institutional study is required. Nevertheless, this study provides robust real-world evidence which is obtained from the large-scale study composed of 958 gastric cancer patients.

## Conclusions

Preoperative NRI is a predictor of overall survival in stage 2 or 3 gastric cancer patients and skeletal muscle loss during the first postoperative year was significantly associated with the prognosis regardless of relapse in stage 3 tumors. These factors could be valuable adjuncts for accurate prediction of prognosis in gastric cancer patients.

## Supplementary Information

**Additional file 1 Supplementary Figure S1.** Web-based toolkit for automatic segmentation of body composition. **Supplementary Figure S2.** Representative cases.

**Additional file 2 Supplementary Table S1.** Changes in body composition and nutritional parameters measured before and 1 year after surgery. **Supplementary Table S2.** Comparison of clinicopathologic characteristics of stage 2 and 3 gastric cancer patients based on NRI and dSMI.

## Data Availability

The datasets used and analysed during this study are available from the corresponding author on reasonable request.

## Abbreviations

AUC: Area under curve
BMI: Body mass index
CI: Confidence interval
CT: Computed tomography
HR: Hazard ratio
NRI: Nutritional risk index
dNRI: difference in nutritional risk index
ROC: Receiver operating characteristic
SMA: Skeletal muscle area
SMI: Skeletal muscle index
dSMI: Difference in skeletal muscle index
SFA: Subcutaneous fat area
VFA: Visceral fat area
