# Supplementary material for: Preoperative nutritional risk index and postoperative one-year skeletal muscle loss can predict the prognosis of patients with gastric adenocarcinoma: a registry-based study
Source: BMC Cancer. 2021 Feb 12;21:157. doi: 10.1186/s12885-021-07885-7 (PMC7881577; doi:10.1186/s12885-021-07885-7)
Supplement: Supplementary file 1 — Additional file 1 Supplementary Figure S1. Web-based toolkit for automatic segmentation of body composition. Supplementary Figure S2. Representative cases. [file 12885_2021_7885_MOESM1_ESM.zip › supplementary figure legendsR4.docx]

**SUPPLEMENTARY FIGURES**

**Supplementary Figure S1. Web-based toolkit for automatic segmentation of body composition**

A CT image is uploaded in a web-based software (assessed at <https://iaidimage.com/app/AID-U/sarcopenia-l3>). Then, a muscle quality map is automatically generated that is composed of the segments; subcutaneous fat area (SFat), visceral fat area (VFat), intermuscular fat (IMA), low-attenuation muscle area (LAMA), and normal-attenuation muscle area (NAMA). The skeletal muscle area (SMA) is calculated by the sum of LAMA and NAMA.

**Supplementary Figure S2. Representative cases**

(A) A 65-year-old female with stage 2 gastric cancer treated with distal gastrectomy and adjuvant chemotherapy. After surgery, the skeletal muscle area (SMA) has been preserved (from 105 to 98), while the visceral fat area (VFA) decreased from 139 to 95 and the superficial fat area (SFA) increased from 81 to 109. She survived longer than five years after surgery without relapse.

(B) A 62-year-old man with stage 3 gastric cancer treated with distal gastrectomy and adjuvant chemotherapy. The SMA, VFA, and SFA markedly decreased during the postoperative one year. As he could not tolerate chemotherapy, a 40% dose reduction was conducted. He died of recurrence including liver metastases three years after surgery.
