# Supplementary figures and images for: Preoperative nutritional risk index and postoperative one-year skeletal muscle loss can predict the prognosis of patients with gastric adenocarcinoma: a registry-based study
Source: BMC Cancer. 2021 Feb 12;21:157. doi: 10.1186/s12885-021-07885-7 (PMC7881577; doi:10.1186/s12885-021-07885-7)

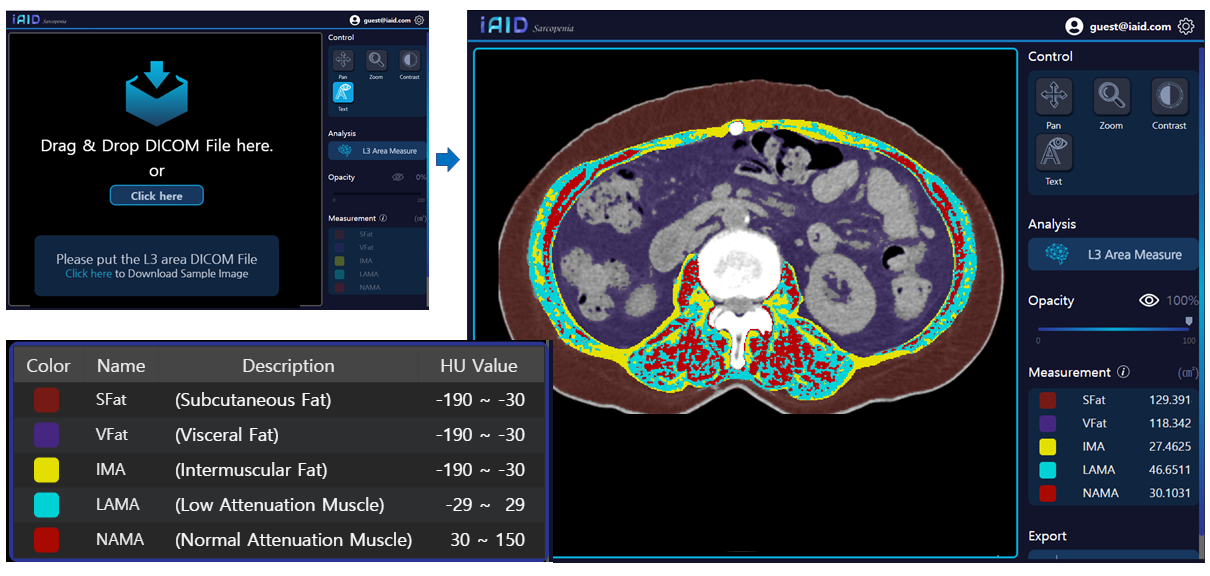

Supplement: Supplementary file 1 — Additional file 1 Supplementary Figure S1. Web-based toolkit for automatic segmentation of body composition. Supplementary Figure S2. Representative cases. [file 12885_2021_7885_MOESM1_ESM.zip › Supplementary Figure S1R4.tiff]

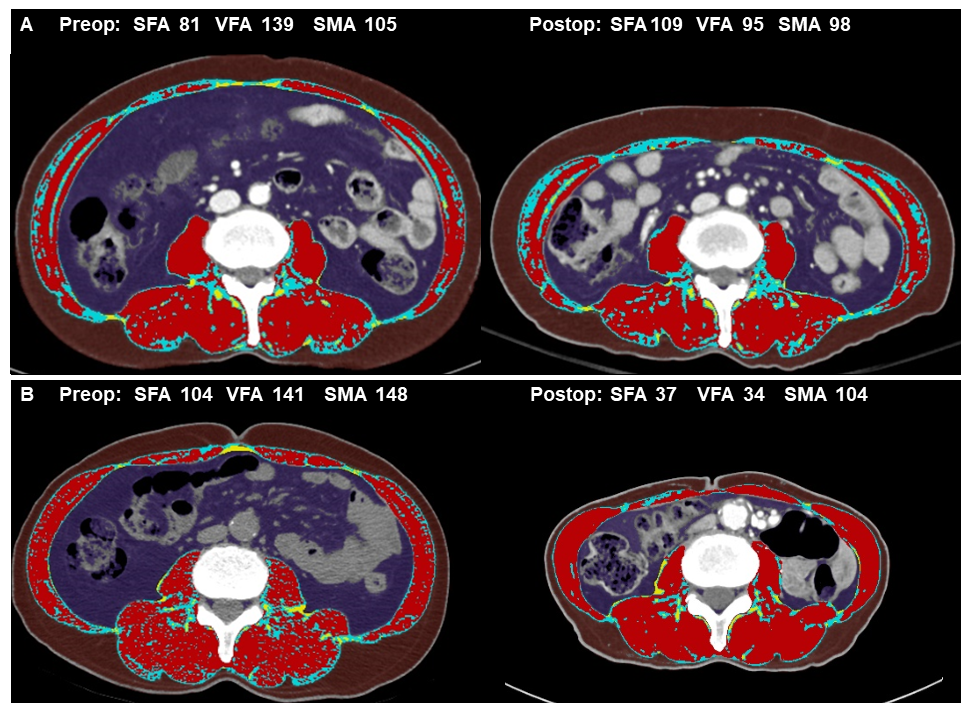

Supplement: Supplementary file 1 — Additional file 1 Supplementary Figure S1. Web-based toolkit for automatic segmentation of body composition. Supplementary Figure S2. Representative cases. [file 12885_2021_7885_MOESM1_ESM.zip › Supplementary Figure S2R4.tif]
