# Supplementary material for: Preoperative nutritional risk index and postoperative one-year skeletal muscle loss can predict the prognosis of patients with gastric adenocarcinoma: a registry-based study
Source: BMC Cancer. 2021 Feb 12;21:157. doi: 10.1186/s12885-021-07885-7 (PMC7881577; doi:10.1186/s12885-021-07885-7)
Supplement: Supplementary file 2 — Additional file 2 Supplementary Table S1. Changes in body composition and nutritional parameters measured before and 1 year after surgery. Supplementary Table S2. Comparison of clinicopathologic characteristics of stage 2 and 3 gastric cancer patients based on NRI and dSMI. [file 12885_2021_7885_MOESM2_ESM.docx]

**Supplementary Table S1. Changes in body composition and nutritional parameters measured before and 1 year after surgery**

| **Variables** | | **Whole patients (N=958)** | | | **Recurrence group (N=293)** | | | **Non-recurrence group (N=665)** | | |
| --- | --- | --- | --- | --- | --- | --- | --- | --- | --- | --- |
| **Category** | **Parameters** | **Pre** | **Post-1Y** | ***P*-value** | **Pre** | **Post-1Y** | ***P*-value** | **Pre** | **Post-1Y** | ***P*-value** |
| Demographic | Weight (kg)  BMI (kg/m^2^) | 62.4 (±10.0)  23.5 (±2.9) | 56.5 (±9.5)  21.2 (±2.6) | **<.001**  **<.001** | 62.1 (±10.0)  23.3 (±3.0) | 55.8 (±9.8)  20.9 (±2.8) | **<.001**  **<.001** | 62.3 (±9.9)  23.4 (±2.8) | 56.9 (±9.3)  21.3 (±2.5) | **<.001**  **<.001** |
| Body composition | SMA (cm^2^)  SMI (cm^2^/m^2^)  SFA (cm^2^)  VFA (cm^2^) | 122.4 (±29.2)  46.6 (±17.0)  113.7 (±57.2)  95.6 (±57.1) | 115.3 (±26.4)  44.1 (±17.0)  73.8 (±47.5)  39.0 (±32.8) | **<.001**  **<.001**  **<.001**  **<.001** | 120.4 (±28.5)  46.4 (±19.4)  111.4 (±59.7)  95.7 (±62.9) | 111.8 (±25.4)  43.1 (±18.4)  70.8 (±48.8)  39.5 (±35.2) | **<.001**  **<.001**  **<.001**  **<.001** | 123.2 (±29.4)  46.7 (±15.8)  114.7 (±56.0)  95.5 (±54.5) | 117.0 (±26.7)  44.6 (±16.2)  75.3 (±46.9)  38.8 (±31.6) | **<.001**  **<.001**  **<.001**  **<.001** |
| Nutritional | NRI  Protein  Albumin | 100.3 (±7.0)  6.7 (±0.6)  3.86 (±0.45) | 93.0 (±14.2)  7.0 (±0.6)  3.95 (±0.42) | **0.003**  **<.001**  **<.001** | 98.9 (±7.5)  6.6 (±0.7)  3.76 (±0.48) | 92.8 (±12.5)  6.9 (±0.7)  3.80 (±0.49) | **0.014**  **0.001**  0.301 | 100.9 (±6.7)  6.7 (±0.6)  3.92 (±0.43) | 93.0 (±14.9)  7.1 (±0.5)  4.02 (±0.37) | **0.010**  **<.001**  **<.001** |
| Abbreviations: Pre, preoperative; Post-1Y, postoperative one year; BMI, body mass index; SMA, skeletal muscle area; SMI, skeletal muscle index, SMA/height^2^; SFA, subcutaneous fat area; VFA, visceral fat area; NRI, nutritional risk index | | | | | | | | | | |

**Supplementary Table S2. Comparison of clinicopathologic characteristics of stage 2 and 3 gastric cancer patients based on NRI and dSMI**

| **Variables** | **NRI (N=958)** | | | **dSMI (N=958)** | | |
| --- | --- | --- | --- | --- | --- | --- |
| **Category** | **Higher NRI:**  **Low-risk group** | **Lower NRI:**  **High-risk group** | ***P*-value** | **Smaller dSMI:**  **Low-risk group** | **Larger dSMI:**  **High-risk group** | ***P*-value** |
| Mean age at operation, years (±SD) | 60.0 (±11.6) | 53.9 (±12.2) | **< 0.001** | 57.4 (±12.3) | 55.4 (±12.1) | **0.018** |
| Sex  Male  Female | 314 (65.6%)  165 (34.4%) | 310 (64.7%)  169 (35.3%) | 0.786 | 278 (58.0%)  201 (42.0%) | 346 (72.2%)  133 (27.8%) | **< 0.001** |
| Charlson Comorbidity Index  0  1-2  3-6 | 158 (33.0%)  235 (49.1%)  86 (17.9%) | 85 (17.8%)  230 (48.0%)  164 (34.2%) | **< 0.001** | 130 (27.1%)  227 (47.4%)  122 (25.5%) | 113 (23.6%)  238 (49.7%)  128 (26.7%) | 0.978 |
| Type of operation  Laparoscopy  Open  Type of gastrectomy  Distal gastrectomy  Total gastrectomy  TNM stage*  2A  2B  3A  3B  3C | 65 (13.7%)  414 (86.4%)  284 (59.2%)  195 (40.8%)  114 (23.8%)  119 (24.8%)  96 (20.0%)  89 (18.6%)  61 (12.8%) | 37(7.8%)  442 (92.2%)  265 (55.3%)  214 (44.7%)  85 (17.7%)  118 (24.6%)  98 (20.5%)  105 (21.9%)  73 (15.3%) | **0.003**  0.215  0.156 | 45 (9.4%)  434 (90.6%)  311 (64.9%)  168 (35.1%)  103 (21.5%)  136 (28.4%)  100 (20.9%)  91 (19.0%)  49 (10.2%) | 57 (11.9%)  422 (88.1%)  238 (49.7%)  241 (50.3%)  96 (20.0%)  101 (21.1%)  94 (19.6%)  103 (21.5%)  85 (17.8%) | **< 0.001**  **< 0.001**  **0.021** |

*TNM stage was based on the American Joint Committee on Cancer 7^th^ edition

NRI and dSMI refer to preoperative nutritional risk index and difference of skeletal muscle index between before- and 1 year after gastrectomy.

Risk group was dichotomized by a median value of each variable.
